# Supplementary material for: Identify hidden spreaders of pandemic over contact tracing networks
Source: Sci Rep. 2023 Jul 19;13:11621. doi: 10.1038/s41598-023-32542-3 (PMC10356757; doi:10.1038/s41598-023-32542-3)
Supplement: Supplementary file 1 — Supplementary Information. [file 41598_2023_32542_MOESM1_ESM.pdf]

Identify Hidden Spreaders of Pandemic over Contact Tracing Networks – Supplementary Information

Shuhong Huang[1], Jiachen Sun[2], Ling Feng[3,4], Jiarong Xie[1], Dashun Wang[5], and Yanqing Hu[6]

[1]School of Data and Computer Science, Sun Yat-sen University, Guangzhou 510006, China
[2]MIT Center for Collective Intelligence, Cambridge 02142, MA, USA
[3]Institute of High Performance Computing, A*STAR, 138632 Singapore
[4]Department of Physics, National University of Singapore, Singapore 117551
[5]Kellogg School of Management, Northwestern University, Evanston, IL, USA
[6]Department of Statistics and Data Science, College of Science, Southern University of Science and Technology, 518055 Shenzhen, China

March 16, 2023

Contents

1 Data **1**
1.1 Empirical COVID-19 spreading data in Singapore . . . 2
1.2 Social networks . . . 2
1.3 The transmission characteristics of COVID-19 . . . 2

2 Simulation of COVID-19 Spreading **3**

3 Machine-Learning-Based Detection Algorithms **3**

4 Containment Strategy **4**

5 Robustness Test **5**
5.1 Fraction of asymptomatic infections, p . . . 5
5.2 Distribution of asymptomatic duration, f_A . . . 5
5.3 Basic reproduction number, R_0 . . . 5
5.4 Length of the state vector, L . . . 5
5.5 Application on another network . . . 5

1 Data

The network data used in this study consist of two parts. The first part is the COVID-19 real spreading data from Singapore, which is used to validate the utility of the proposed method. The other part contains two real social network structures, on which we simulate COVID-19 spreading and access the control effect of different screening strategies.

1.1 Empirical COVID-19 spreading data in Singapore

COVID-19 data of infected patients in Singapore [1, 2] is obtained from the website of Ministry of Health of Singapore, in which nodes correspond to real-world entities, including confirmed infections and public places (e.g., hospital, aircraft) where the infections have visited, while links correspond to the contact relationship between the nodes. In addition to tracing the human-to-human contact, the dataset also constructs contact relationship based on the geographical location of patients. Specifically, the original Singapore data uses the connections of public places where the exposure occurred as a proxy for the contact of patients. In other words, if a patient has visited a place, a link is created between the patient and the place. In this study, we regard public places as common nodes that can infect and spread viruses, so as to simulate the spread of COVID-19. In particular, we extract two disconnected components in the whole network, leading to two independent COVID-19 contract-tracing networks, denoted by Singapore A and Singapore B, respectively (see in Fig.2 c,d in the main text). Singapore A contains 35 nodes (3 of them are public places) and 56 links, in which the symptoms date is from January 23 to February 21. Singapore B contains 65 nodes (3 of them are public places) and 109 links, in which the symptoms date is from February 11 to March 21.

1.2 Social networks

- **Infectious Stay Away Network (ISA)**: We use a contact network from the exhibition "Infectious: Stay Away" [11] which was held in Dublin on 15 July 2009. The network is download from http://konect.cc/netw orks/sociopatterns-infectious/, containing 410 nodes (individuals) and 2847 links. Each of links denotes the existence of face-to-face communication between two nodes during the exhibition, which is analogous to the way COVID-19 is transmitted in the population.
- **Email Network**: The email communication network of University of Rovira i Virgili [8] is obtained from http://networkrepository.com/email-univ.php. Nodes in network are different members of the school. If member i sent at least one email to member j, the network contains a directed edge from i to j. In this work, we ignore the direction of links by using undirected links to represent the existence of communication between nodes, which implies that people who communicate via email in college are also likely to have contact in offline life. The network contains 1133 nodes and 5451 links.

1.3 The transmission characteristics of COVID-19

The epidemiological characteristics and clinical parameters of COVID-19 used in this study are introduced as follows.

- **Basic reproduction number** R_0: The value of R_0 used in this work is 3.5, which is the average from 10 empirical studies of COVID-19 [6, 10, 14, 15, 18–21, 23, 25] published from 7 January 2020 to 7 February 2020.
- **Incubation period distribution of presymptomatic infections** $f_P(d)$: According to the clinical data published in [13], we fit $f_P(d)$ by a log-normal distribution with $\mu_P = 0.62$ and $\sigma_P = 0.64$.
- **Distribution of Infection duration** $f_I(d)$: For symptomatic COVID-19 patients, we adopt a normal distribution with $\mu_I = 8.8$ and $\sigma_I = 3.88$ to fit $f_I(d)$ according to [13].
- **Fraction of asymptomatic infection,** p: We set $p = 15\%$ in the main text, which is the minimal value from [4, 5, 16, 17, 17, 24] (15%-80%). We also consider other values of p and discuss the corresponding performances in Sec. SI 5.

- **Incubation period distribution of asymptomatic infections, $f_A(d)$**: Generally, an asymptomatic patient has no access to know the exact time of being infected and being recovered. In the main text, we used the time from the onset of the disease to the time the virus become no longer detectable [26] as the average duration $\mu_A = 20$ for asymptomatic infected persons to carry the virus. Based upon this, we approximate $f_A(d)$ by a normal distribution with $\sigma_A = 5$. We discuss other values of μ_A and σ_A in the Sec. SI 5.

- **COVID-19 spreading probability β**: In this work, the spreading probability β is inferred based on the fact that an infected individual could infect R_0 people in average. Specifically, we define λ as the average time a susceptible person carries the virus:

$$\lambda = p \cdot \mu_A + (1-p) \cdot (\exp(\mu_P + \frac{\sigma_P^2}{2}) + \mu_I) \tag{1}$$

  Based on this, β is given by:

$$\beta = \frac{R_0}{\langle k \rangle \cdot \lambda} \tag{2}$$

  where $\langle k \rangle$ is the average number of contacts per person (i.e. average degree) in the network.

2 Simulation of COVID-19 Spreading

In this section, we introduce in detail the simulation process of COVID-19 transmission. The spreading model of COVID-19 in this study is similar to the classic Susceptible-Exposed-Infectious-Recovered model [3], with *two* major differences. First, we introduce two latent states, namely, Asymptomatic and Presymptomatic. When a susceptible node becomes infected, it's state either turns to Asymptomatic with the given probability p or turns to Presymptomatic with $1-p$. Second, An infected node (i.e., P, A and I) will last a certain number of days in the current state, determined by the specific probability distribution. Based on the above rules, we update each node's state by using the prior states of each node in the network. Specifically, the node in the infected state (P,A and I) will infect its susceptible neighbor with the probability β and will move to the next state after spending the number of days in the current state. While nodes in the R state will not change to a new state anymore. Fig. S6 illustrates how the nodes are initialized and updated at each day in the simulation.

3 Machine-Learning-Based Detection Algorithms

In this section, we give a description of 4 screening baselines used in this study, including a random screening method (i.e. the average proportion of infections) and three machine-learning based algorithms. The random screening method is simply shuffling all unknown individuals in the network and then using the shuffled list as the screening order. Regarding the machine-learning-based algorithms, we model the quantification of infection probability as a binary classification problem (i.e., infected vs. uninfected), where the training set includes individuals with known state (including the symptomatic infections and the recovered ones), while the testing set includes the unknown nodes in the network. In the testing process, we denote the model's softmax output between [0,1] for each testing sample as it's infected probability. After that, we sort the testing samples according to infected probability in descending order, resulting to the screening order. Below we introduce each machine-learning-based-algorithm in detail.

- **Graph Attention Network (GAT)** [22]: A graph attention network with four layers is employed to learn the infected-vs-uninfected classification problem. Since there is no nodal feature, we use a $N \cdot N$ identity matrix as the node's feature input. The first hidden layer is an Attention layer with 3 head attentions and 24-dimension output. The second hidden layer is also an Attention layer with 1 head attentions and 2-dimension output. The log softmax is used as the loss function at

the output layer. In the training process, the activation function is the leaky ReLU with learning rate 0.005 and $\alpha = 0.2$. To prevent over-fitting, we employ dropout with keep probability 0.6. The training process terminates if the current loss is less than the subsequent 50 consecutive losses.

- **Graph Convolutional Network (GCN)** [12]: We use a four-layers graph convolutional network in this work. Similar to GAT, the input layer is the node's feature represented by a $N \cdot N$ identity matrix. The first and second hidden layers are graph convolutional layers with output-size 16 and 2, respectively. The log softmax is used as the loss function at the output layer. In the training process, we use learning rate 0.05 and introduce a random bias in each layer's output as an activation function. We also employ dropout with keep probability 0.5. The training process terminates after 200 iterations.

- **Node2Vec+MLP** [7]. We generate a 64-dimension vector for each node through Node2vec embedding, where a node is performed 200 independent random-walks, each of which involves 30 nodes in the network. Then we input the generated embedding to a perception with two hidden layers (each of which has 64 dimensions) to learn the infected-vs-uninfected classification problem. In the training process, the activation function is the leaky ReLU with learning rate 0.005 and $\alpha = 0.2$. To prevent over-fitting, we employ dropout with keep probability 0.6. The training process terminates if the current loss is less than the subsequent 50 consecutive losses.

For the above algorithms, we use the source codes published by the original authors and conduct an optimization for the model's parameters. We choose parameters that work well within our parameter selection range.

4 Containment Strategy

In this section, we introduce three different strategies to contain COVID-19 spreading.

- **Infection containment**: Individuals who develop symptoms of COVID-19 (i.e., node's state is Symptomatic) at time t will be quarantined immediately at the following time $t + 1$ (i.e., set its state to Recovered). While the remaining individuals in the network continue to participate in the subsequent COVID-19 propagation.

- **Neighbor containment**: Apart from quarantining symptomatic infections, we also select a random subset of the closest neighbors of known infected individuals for testing. If the COVID-19-test result of a node is positive, it will be quarantined immediately and its neighbors will be added for testing. Given that the practical testing capacity cannot cover all contacts of infected persons, we randomly choose a f fraction of the size of the whole network (i.e., $f \cdot N$) among the neighbors for testing. We present the performance of $f = 2\%$ and $f = 4\%$ in Fig. 3 in the main text and Fig. S18-S19, respectively.

- **Dynamic containment (our method)**: Apart from quarantining symptomatic infections, the proposed method tries to identify and block potential risky individuals by considering both the node's infected probability and its spreading power in the network. Specifically, we define $H_j(t)$ as the infectious risk indicator for node j at time t, which is equal to the product of the probability that j is infected (i.e., in P and A) and the ability of j to infect its closest [9] susceptible neighbors:

$$H_j(t) = (P_j(t) + A_j(t)) \cdot (1 + \beta \cdot \sum_{i \in \partial j} S_i(t)) \tag{3}$$

At time t, we sort the unknown individuals in the network with $H_j(t)$ in descending order and then choose the top $f \cdot N$ nodes for testing, where N is the network size. Similar to the Neighbor contain, if the COVID-19 test result of a node is positive, it will be quarantined immediately and

its neighbors will be added into the testing set. Note that the screened nodes will be added into the training set and help to infer other unknown nodes' states in the following days.

5 Robustness Test

In this section, we conduct robustness analysis for the proposed screening method. In particular, we study COVID-19 parameters that may vary in the actual spreadings. These parameters include the fraction of asymptomatic infections p, the incubation period distribution of asymptomatic infections f_A, the cut-off dimension of state vector L, the basic reproduction number R_0 and the deviation in the exact time of infection. The above parameter's sensitivity are tested on the ISA network used in the main text.

Besides, we also investigate the effect of network structure by applying the proposed method to an email network (see Sec. SI 5.5). At $T = 0$, we choose 5 individuals in random as initial spreaders in the network. From $T = 10$, we start inferring the infected probability of the unknown nodes based on the information of the known symptomatic infections.

5.1 Fraction of asymptomatic infections, p

Fig. S7 presents the accuracy and recall of the proposed method using $p = 0.15, 0.30$ and 0.45. One can observe that the accuracy of the algorithm can be influenced by a high value of p on the closest-neighbor sub-network. Overall the performance remains almost unchanged on the global network.

5.2 Distribution of asymptomatic duration, f_A

The screening performances of the proposed algorithm on the mean μ_A and on the derivation σ_A are shown on Fig. S8 and Fig. S9, respectively. The result shows our method is robust to different distribution of asymptomatic duration.

5.3 Basic reproduction number, R_0

Fig. S10 presents the accuracy and recall of the proposed method using $R_0 = 2, 3.5$ and 5. Although the values of accuracy vary across the three R_0 due to the difference in the proportion of infected people in the network, the qualitative findings on the recall rate are almost identical.

5.4 Length of the state vector, L

We measure the effect of cut-off length L of state vector on our algorithm's performance. The lengths for P, A, I are 10, 10, 25 for $L = 47$, and 15, 15, 30 for $L = 62$. As shown in Fig. S11, we can see that both the accuracy and the recall are not sensitive to L. Since the probability of two people who are in continuously close contact being infected in the n-th day decays exponentially with the time of infection, L is relatively robust in this case.

5.5 Application on another network

In the main text, the proposed method is only tested on the ISA network. In this subsection, we apply our method in another typical social network, namely, the email network (see description in Sec. SI 1). Specifically, Figs. S12-S14 present the performance of the proposed algorithm in comparison of machine-learning-based algorithms, under 1-step-neighbor sub-network, 2-step-neighbor sub-network and the whole network, respectively. Fig S15 presents the dynamic screening performance on the email network. Fig S16 presents the horizontal comparison of the accuracy and recall of the proposed screening algorithm under different network structures. Our method's robustness in the incomplete email network is shown in Fig S17. The COVID-19 contain effects are presented in Fig S18.

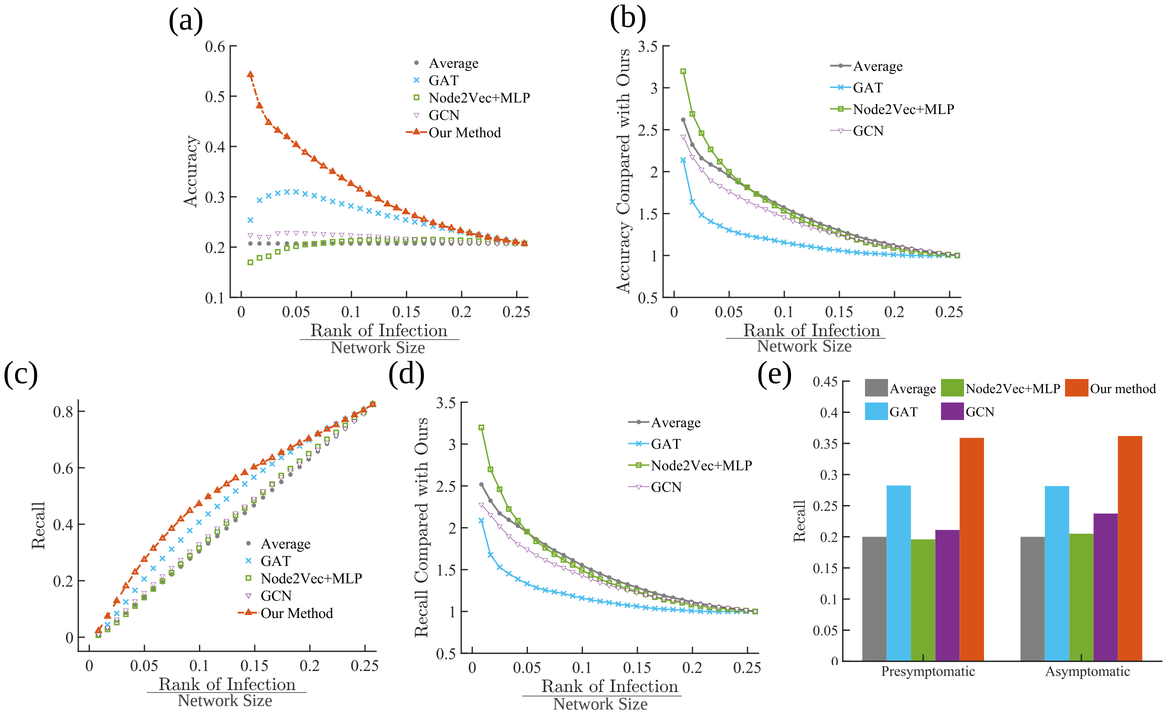

Figure S1: Performance of the static screening method on the 1-step sub-network of ISA network. (a) The accuracy vs. rank of infection (divided by the network size). (b) the ratio between the accuracy of our proposed algorithm and other algorithms. (c) recall rate (d) the relative recall rate of the machine-learning-based algorithms. (e)The recall of asymptomatic patients in the two categories (Presymptomaic and asymptomatic) of the top 20% of this sub-network.

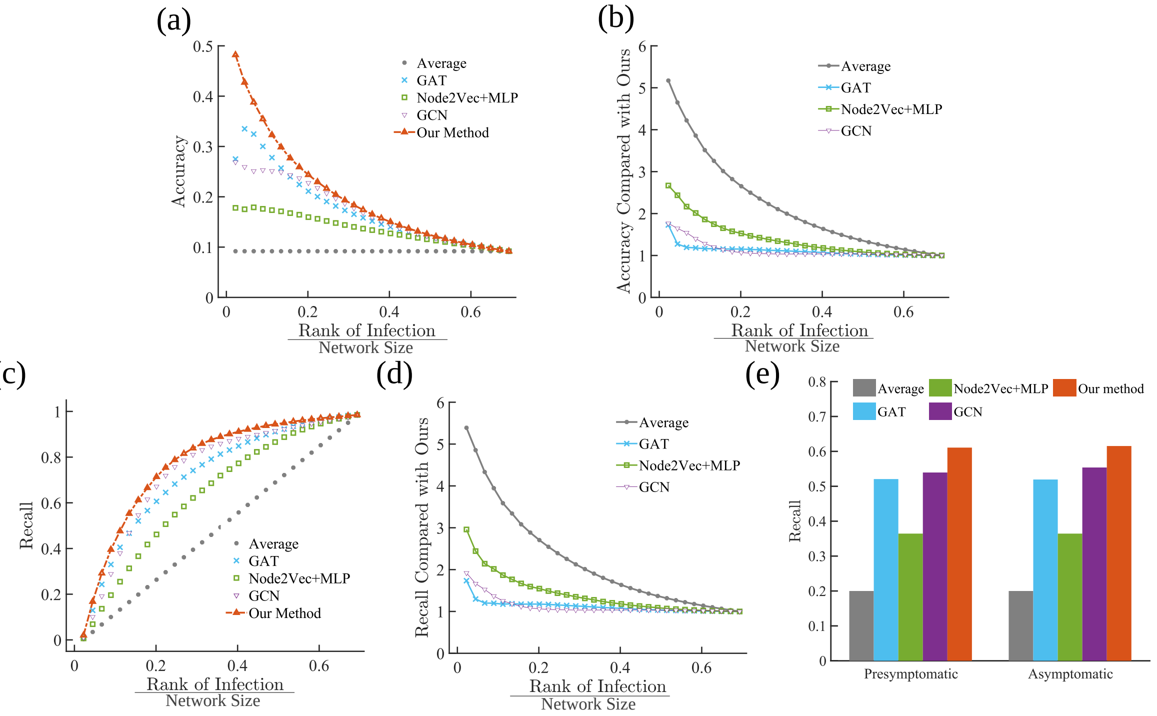

Figure S2: Performance of the static screening method on the 2-step sub-network of ISA network. (a) The accuracy vs. rank of infection (divided by the network size). (b) the ratio between the accuracy of our proposed algorithm and other algorithms. (c) recall rate (d) the relative recall rate of the machine-learning-based algorithms. (e)The recall of asymptomatic patients in the two categories (Presymptomaic and asymptomatic) of the top 20% of this sub-network.

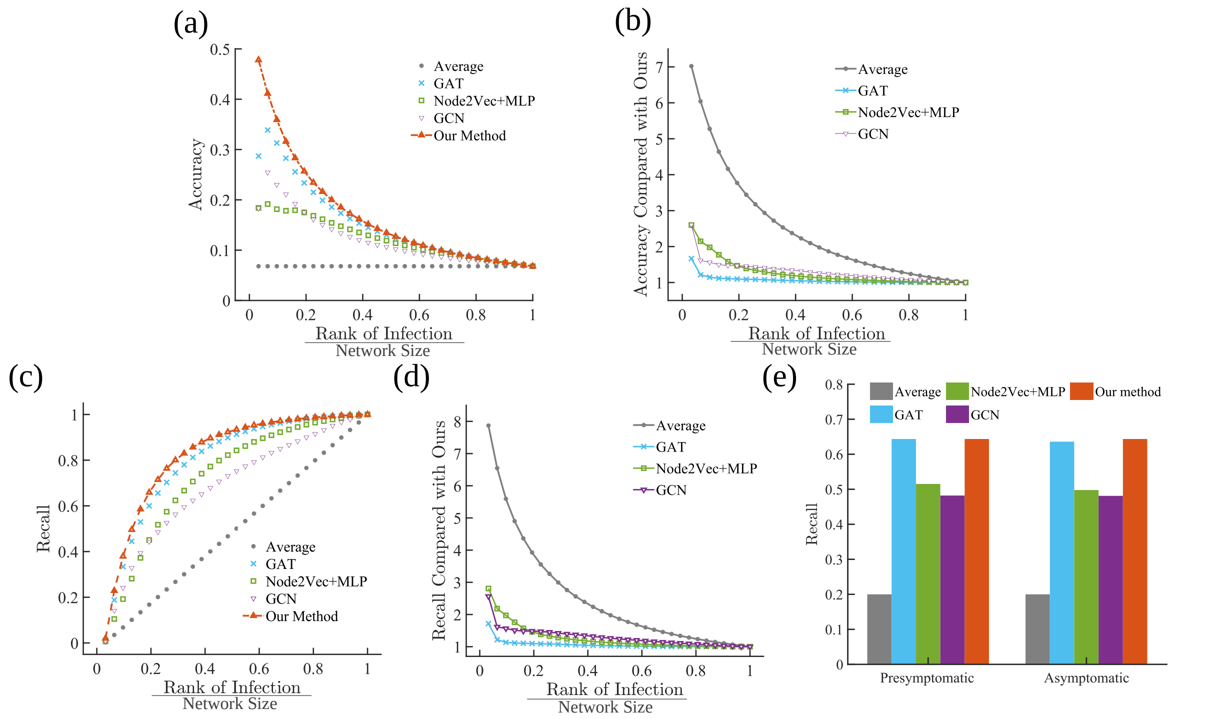

Figure S3: Performance of the static screening method on the whole ISA network. (a) The accuracy vs. rank of infection (divided by the network size). (b) the ratio between the accuracy of our proposed algorithm and other algorithms. (c) recall rate (d) the relative recall rate of the machine-learning-based algorithms. (e)The recall of asymptomatic patients in the two categories (presymptomaic and asymptomatic) of the top 20% of this network.

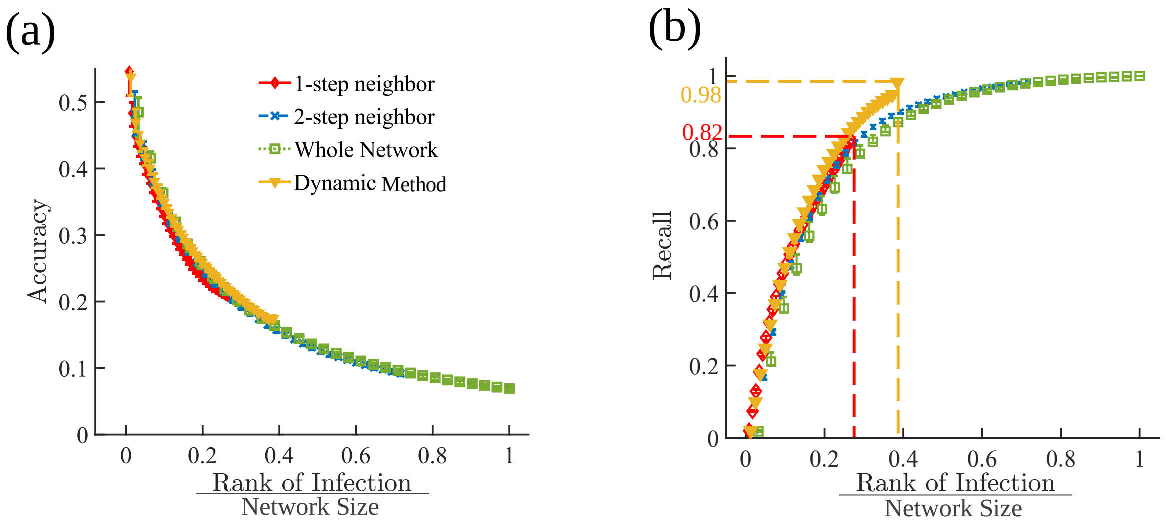

Figure S4: Recall rate of the static algorithms on the 1-step neighbor subnetwork, 2-step neighbor subnetwork and on the whole network, compared with the dynamic algorithm, on the ISA network. Two dash lines in (b) correspond to the ultimate screening number achieved by the dynamic method and the static method on 1-step neighbor, respectively.

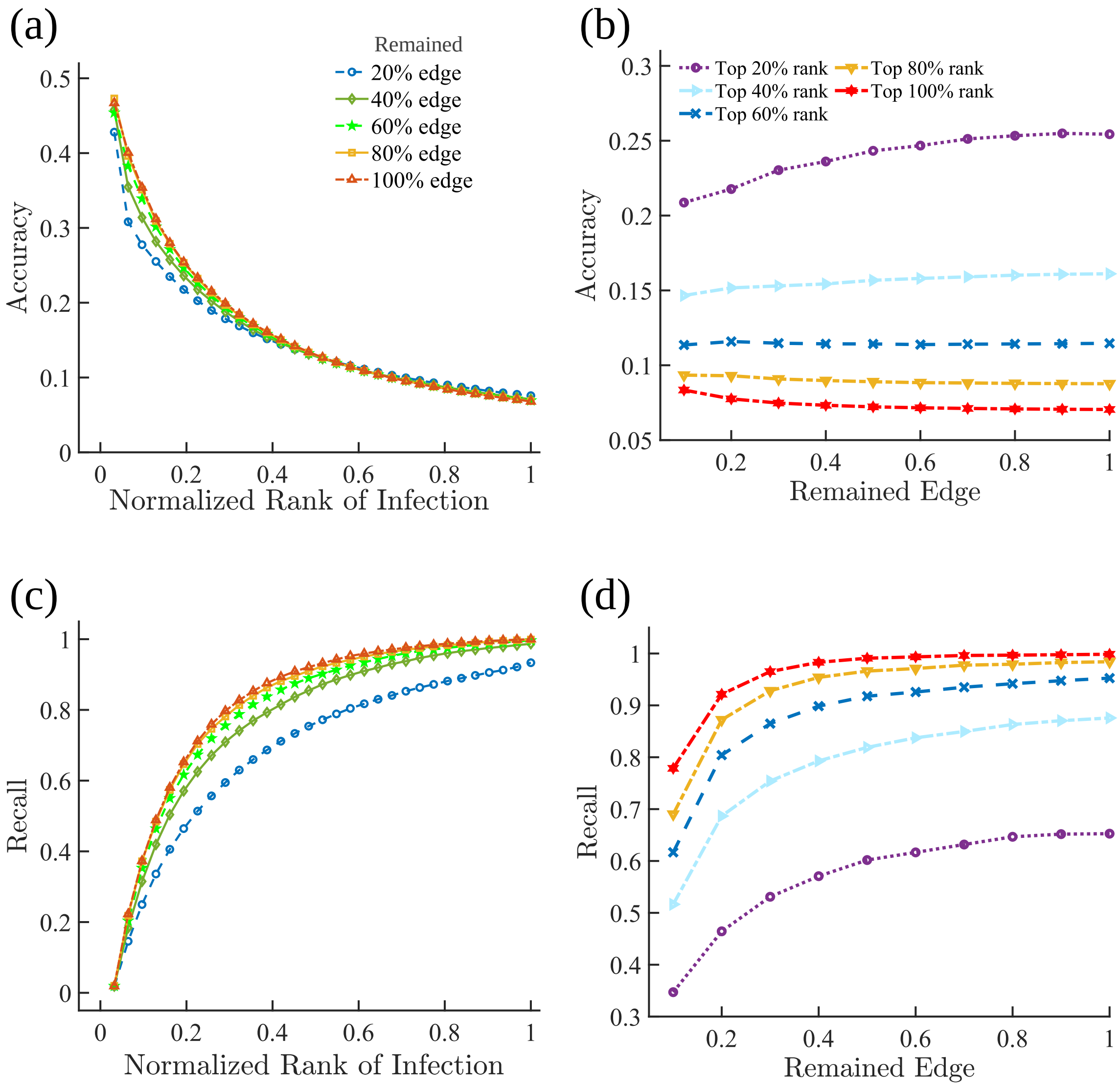

Figure S5: Performance of the static screening method with incomplete network information on the whole ISA network. We randomly remove a fraction of links in the ISA network and then employ the proposed screening schemes on the remaining network. (a) The relationship between the accuracy and the ranking value of the infection probability with different proportions of the removed edges. (b) Accuracy of the dynamic screening method vs. the proportion of the removed edges by measuring the infection rank with different proportions. (c) The dependencies of recall rate of the whole-network screening on the infection rank. (d) Recall vs. the remaining edges.

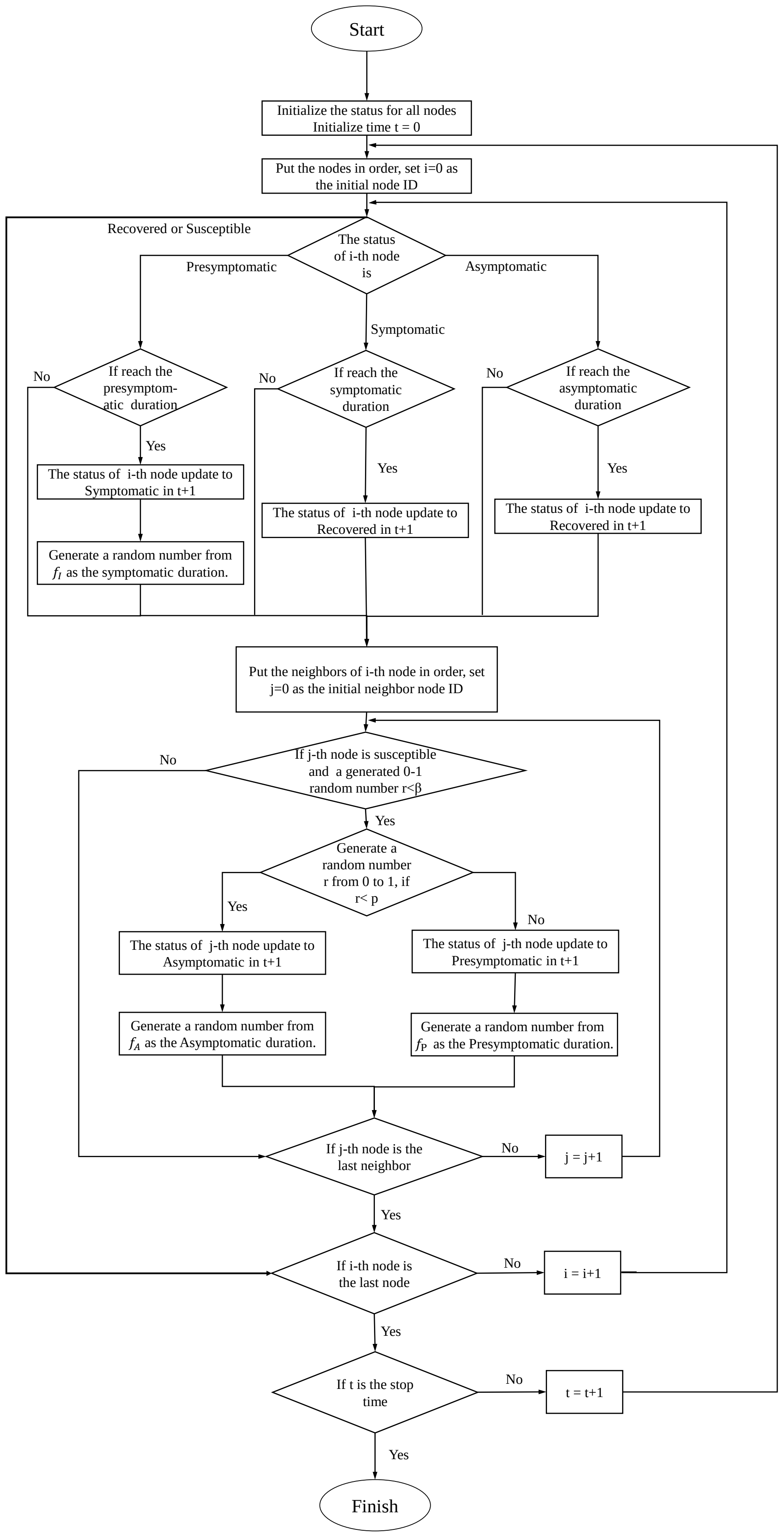

Figure S6: Flowchart of the simulation of COVID-19 transmission on networks.

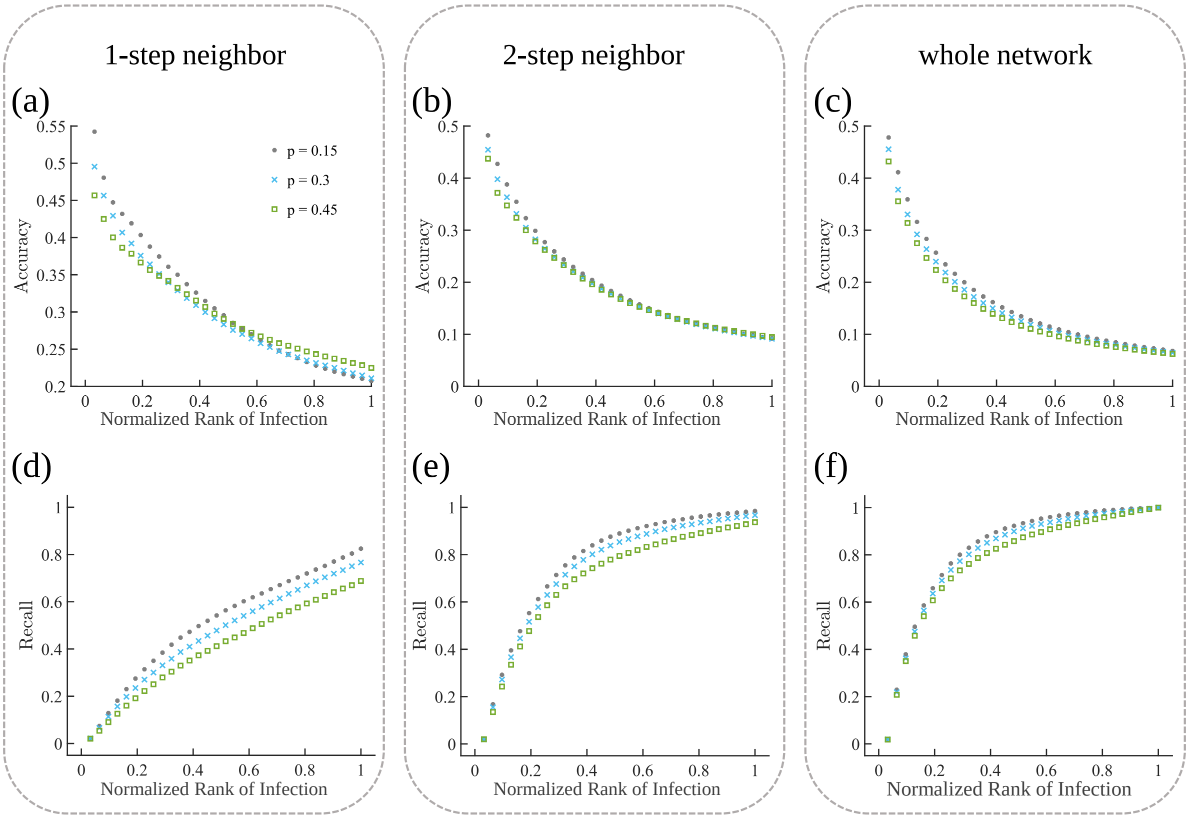

Figure S7: The performance of the proposed static screening method with varying fraction of asymptomatic infections p. (a), (b)and (c) respectively represent the accuracy on the sub-network composed of known infected persons and their neighbors, 2-steps neighbors, and the whole network. (d), (e) and (f) respectively shows the recall.

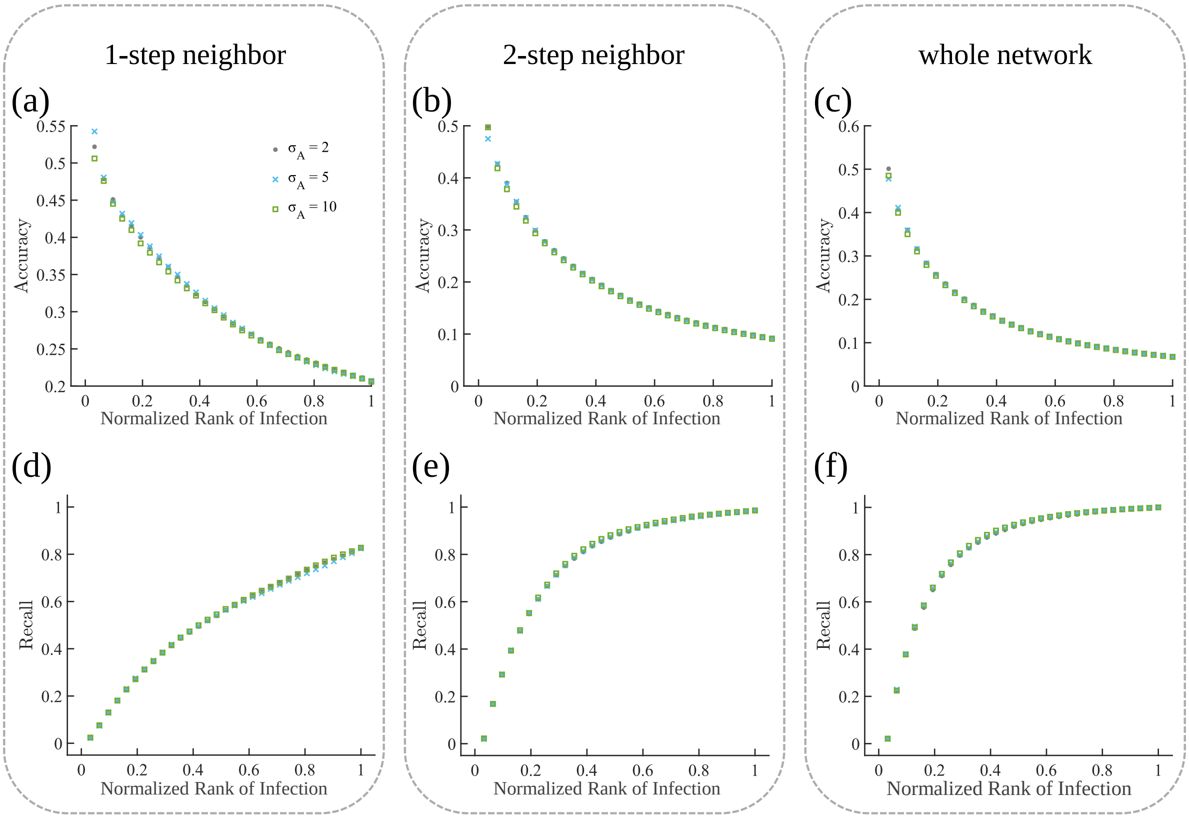

Figure S8: The performance of the proposed static screening method with different standard deviation μ_A for infected persons in asymptomatic state. (a), (b)and (c) respectively represent the accuracy on the sub-network composed of known infected persons and their neighbors, 2-step neighbors, and the whole network. (d), (e) and (f) respectively shows the recall.

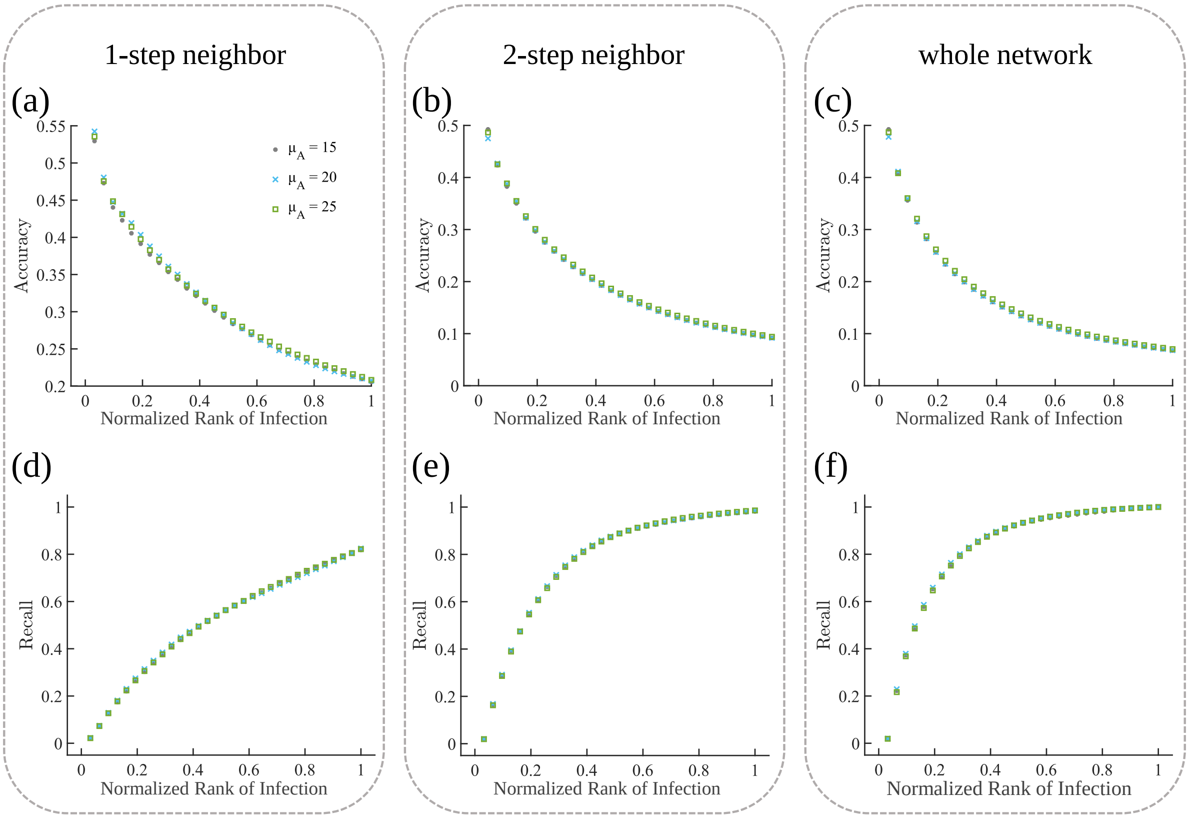

Figure S9: The performance of the proposed static screening method with different average time μ_A for infected persons in asymptomatic state. (a), (b)and (c) respectively represent the accuracy on the sub-network composed of known infected persons and their neighbors, 2-steps neighbors, and the whole network. (d), (e) and (f) respectively shows the recall.

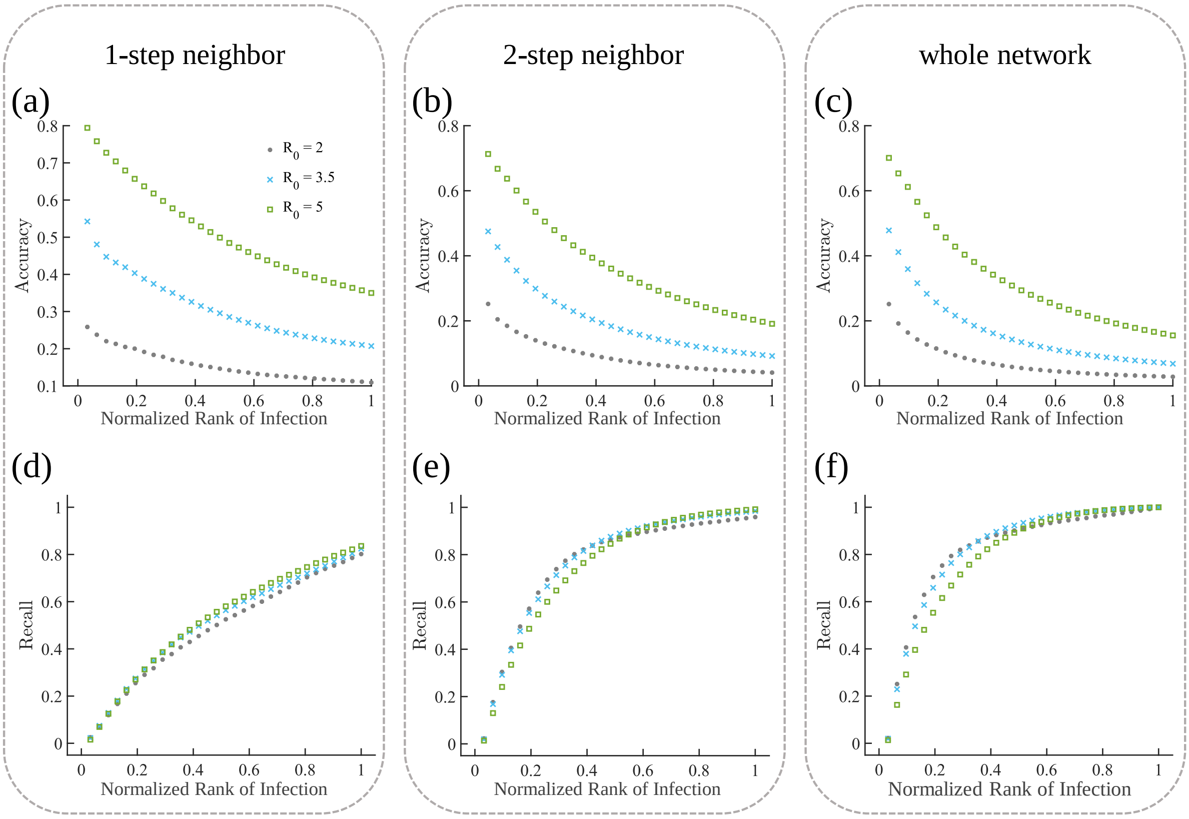

Figure S10: The performance of the proposed static screening method with different basic reproduction number R_0. (a), (b)and (c) respectively represent the accuracy on the sub-network composed of known infected persons and their neighbors, 2-steps neighbors, and the whole network. (d), (e) and (f) respectively shows the recall.

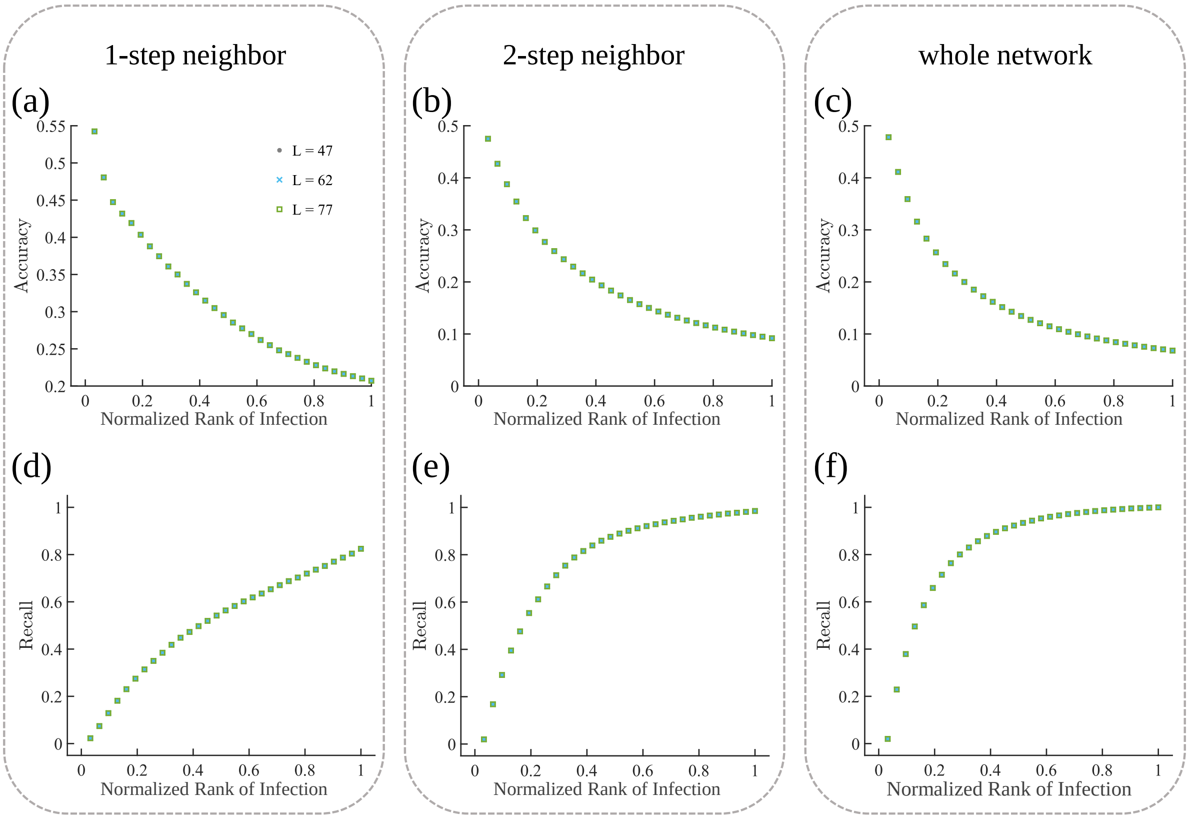

Figure S11: The performance of the proposed static screening method with different vector length L. (a), (b)and (c) respectively represent the accuracy on the sub-network composed of known infected persons and their neighbors, 2-steps neighbors, and the whole network. (d), (e) and (f) respectively shows the recall.

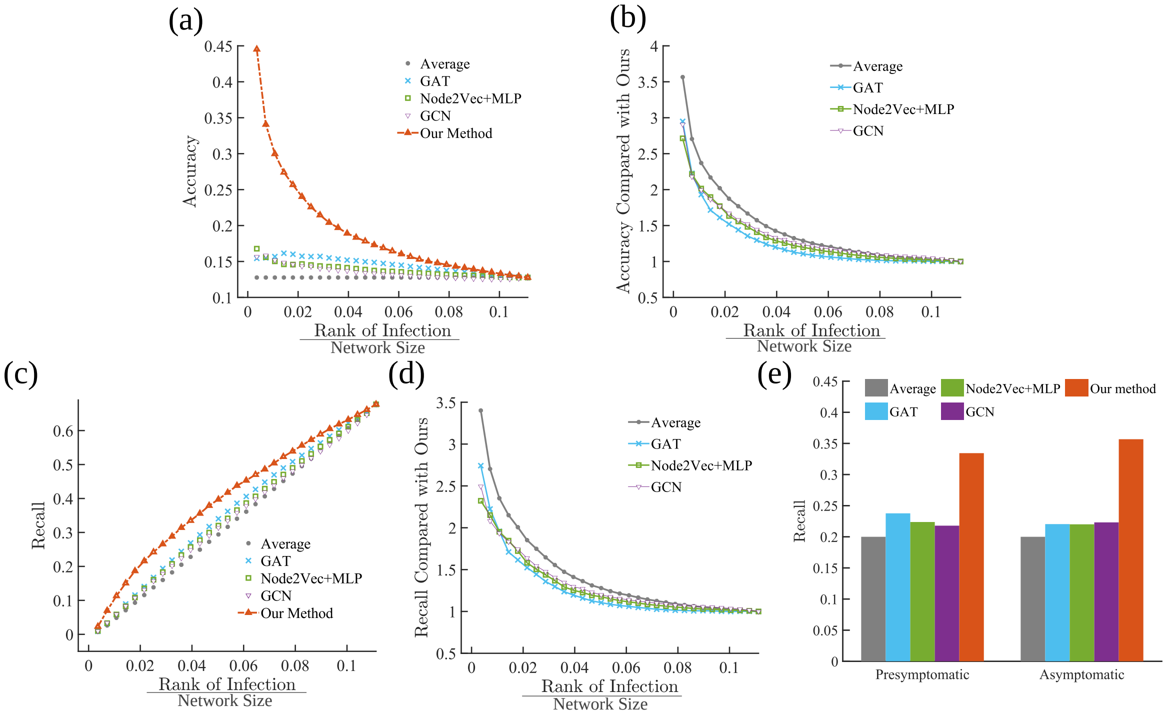

Figure S12: Performance of the static screening method on the 1-step sub-network of Email network. (a) The accuracy vs. rank of infection (divided by the network size). (b) the ratio between the accuracy of our proposed algorithm and other algorithms. (c) recall rate (d) the relative recall rate of the machine-learning-based algorithms. (e)The recall of asymptomatic patients in the two categories (Presymptomaic and asymptomatic) of the top 20% of this sub-network.

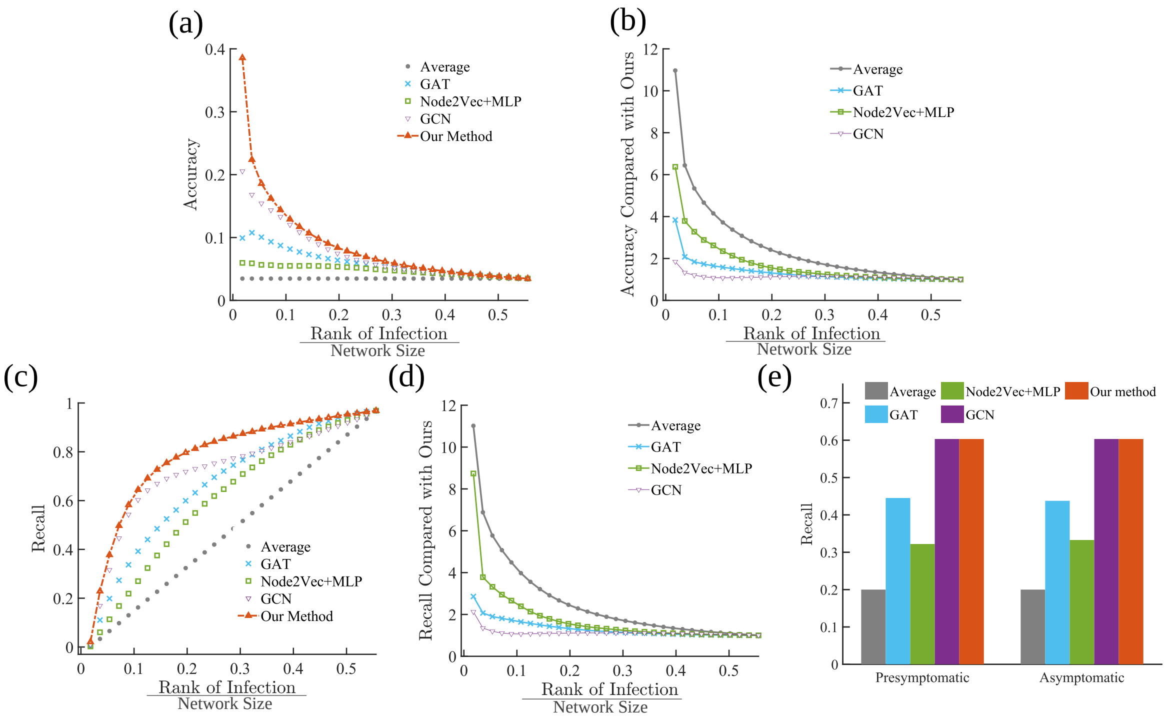

Figure S13: Performance of the static screening method on the 2-step sub-network of Email network. (a) The accuracy vs. rank of infection (divided by the network size). (b) the ratio between the accuracy of our proposed algorithm and other algorithms. (c) recall rate (d) the relative recall rate of the machine-learning-based algorithms. (e)The recall of asymptomatic patients in the two categories (Presymptomaic and asymptomatic) of the top 20% of this sub-network.

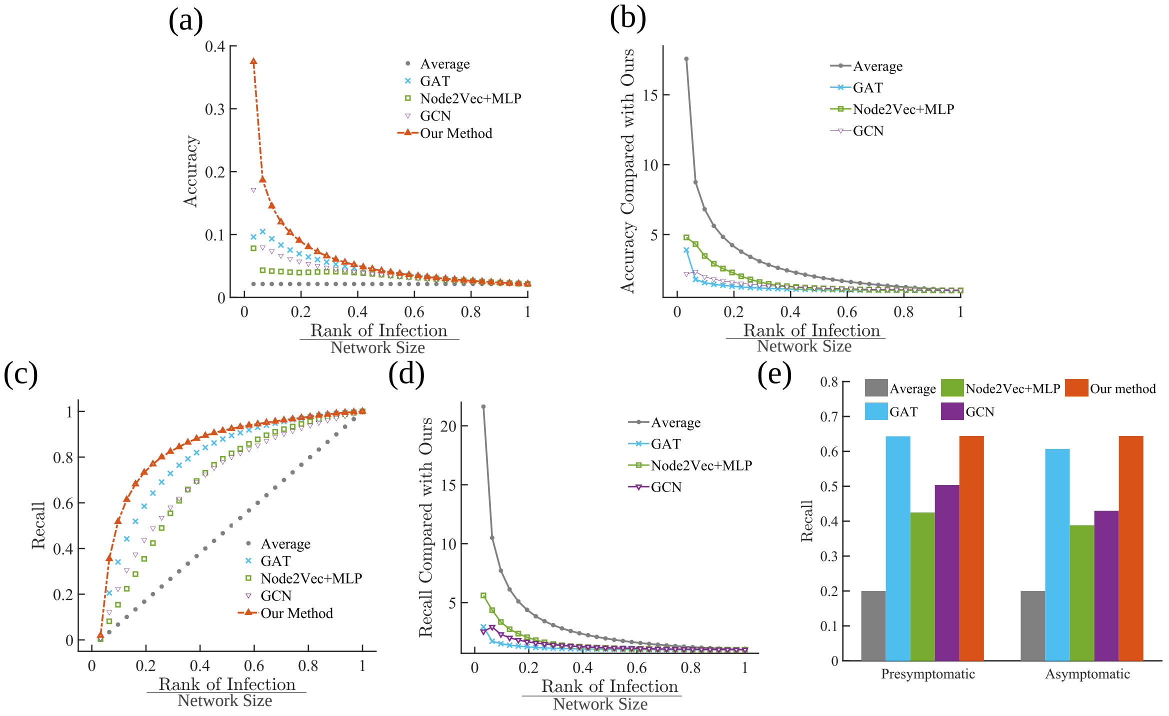

Figure S14: Performance of the static screening method on the whole Email network. (a) The accuracy vs. rank of infection (divided by the network size). (b) the ratio between the accuracy of our proposed algorithm and other algorithms. (c) recall rate (d) the relative recall rate of the machine-learning-based algorithms. (e)The recall of asymptomatic patients in the two categories (Presymptomaic and asymptomatic) of the top 20% of this network.

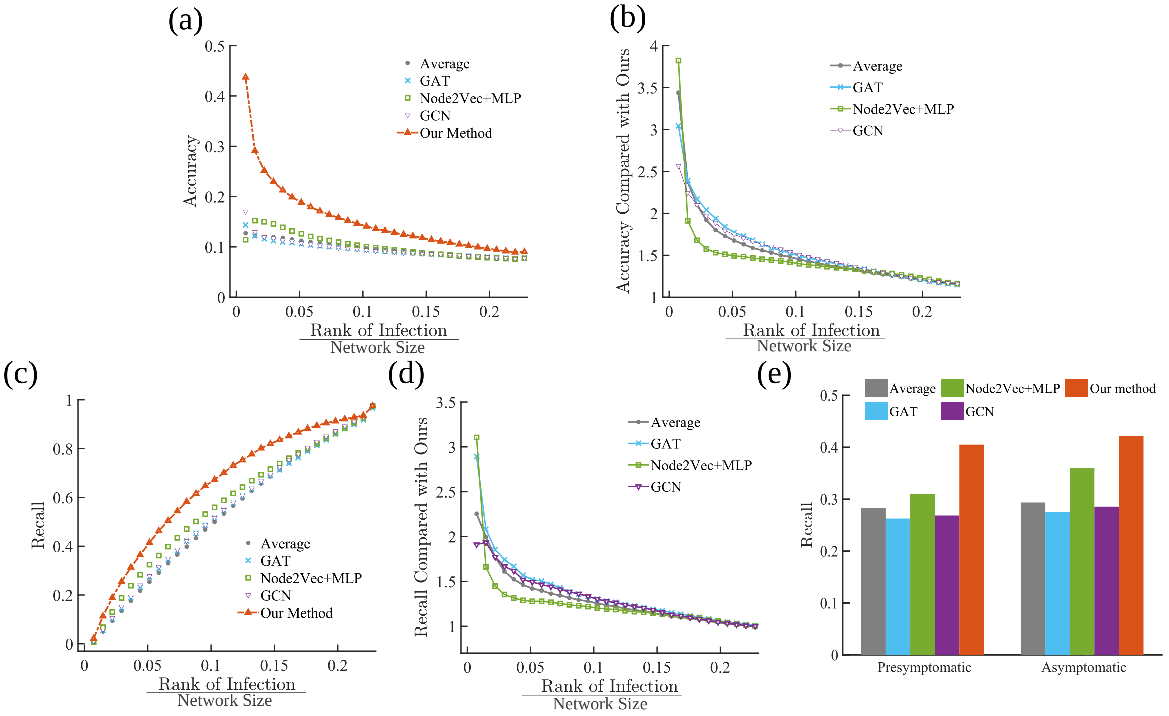

Figure S15: Performance of the dynamic screening method on the Email network. (a) The accuracy vs. rank of infection (divided by the network size). (b) the ratio between the accuracy of our proposed algorithm and other algorithms. (c) recall rate (d) the relative recall rate of the machine-learning-based algorithms. (e)The recall of asymptomatic patients in the two categories (Presymptomaic and asymptomatic) of the top 20% of this network.

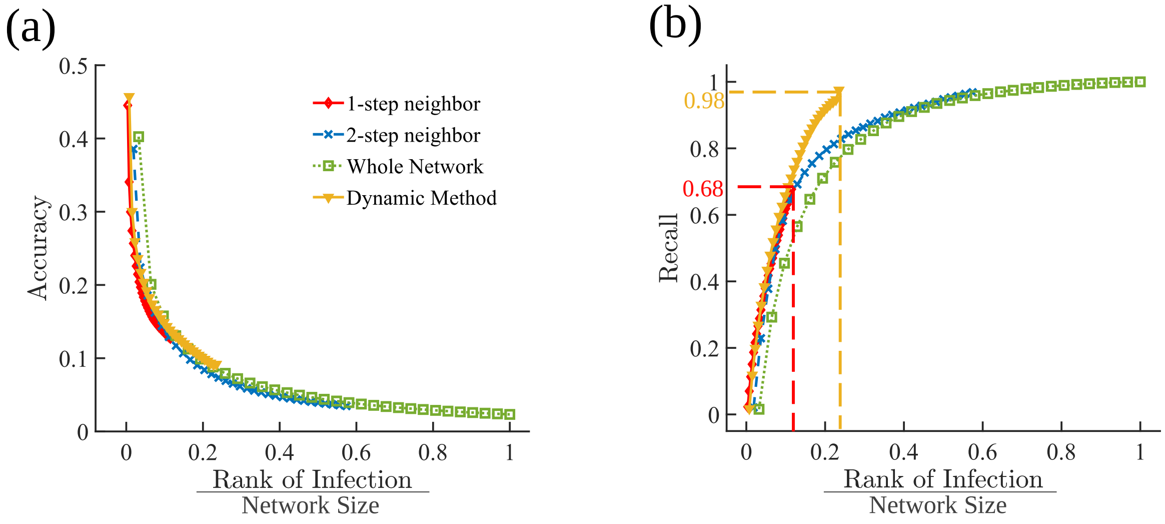

Figure S16: Recall rate of the static algorithms on the 1-step neighbor subnetwork, 2-step neighbor subnetwork and on the whole network, compared with the dynamic algorithm, on the Email network. Two dash lines in (b) correspond to the ultimate screening number achieved by the dynamic method and the static method on 1-step neighbor, respectively.

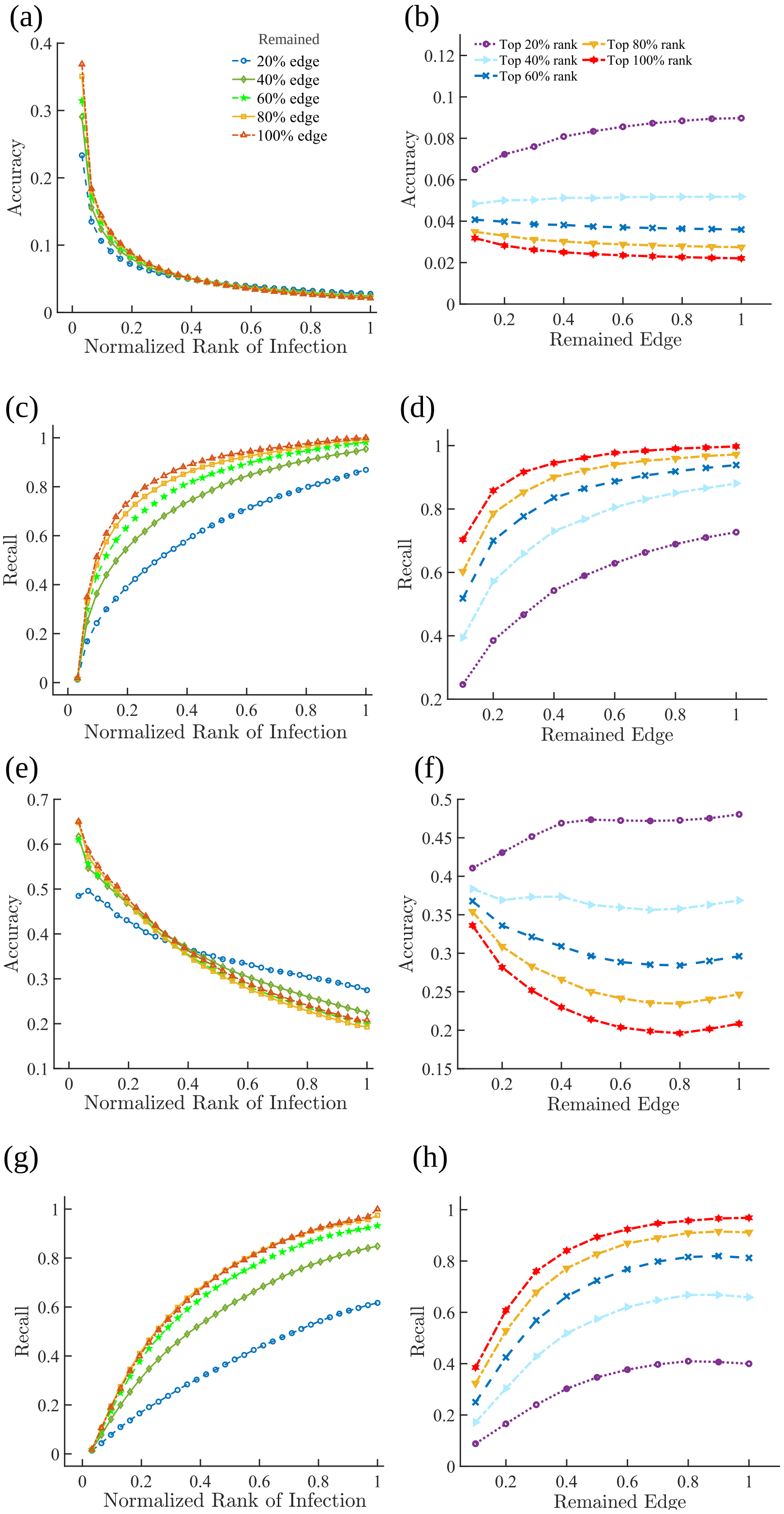

Figure S17: Performance with incomplete network information on the whole Email network. (a-d) Performance of the static screening method (a) The relationship between the accuracy and the ranking value of the infection probability with different proportions of the removed edges. (b) Accuracy of the dynamic screening method vs. the proportion of the removed edges by measuring the infection rank with different proportions. (c) The dependencies of recall rate of the whole-network screening on the infection rank. (d) Recall vs. the remaining edges. (e-h) Performance based on the dynamic screening method.

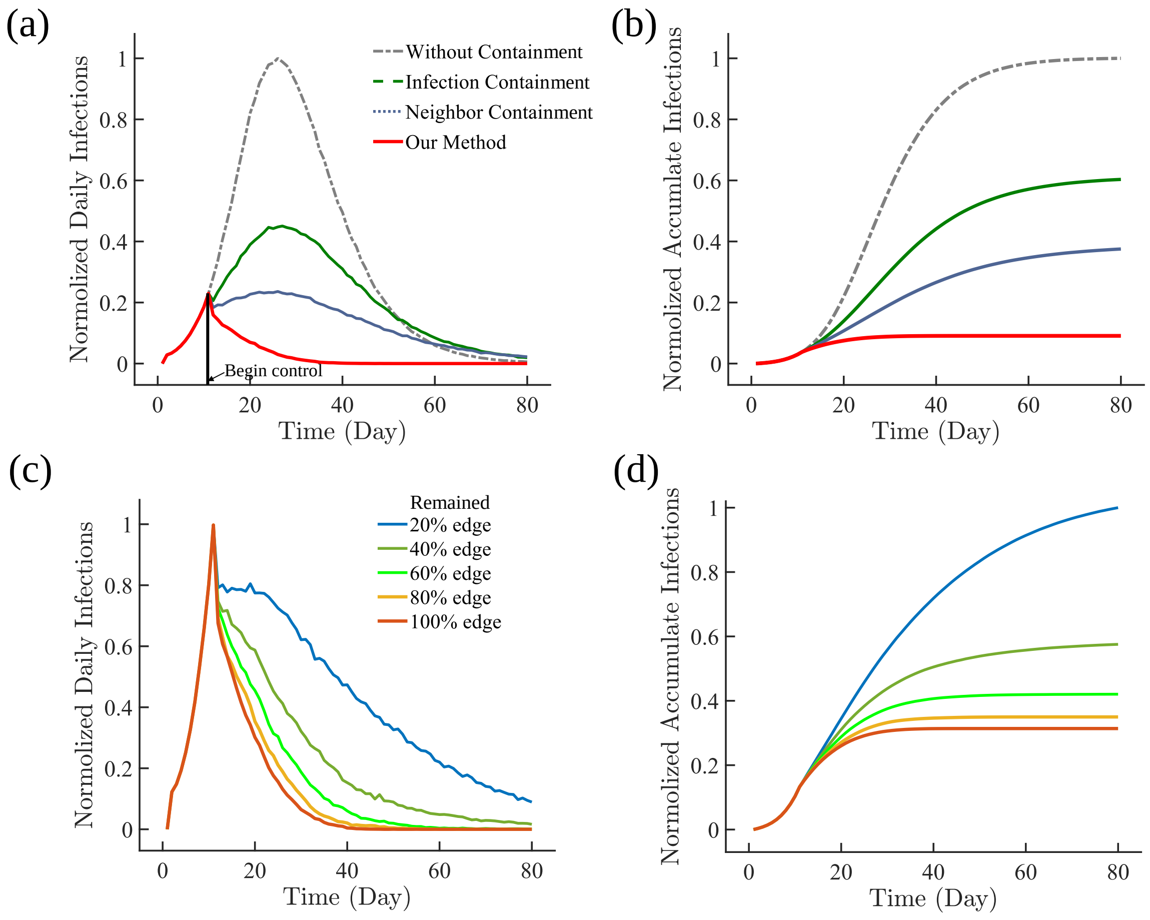

Figure S18: Containment effectiveness of the proposed method on simulated COVID-19 spreading. From $T = 10$ of COVID-19 spreading simulation on the Email network, we conduct different approaches separately to contain the pandemic. For the various containment method, we select a total of 2%N of individuals to screen. (a) The normalized number of daily new infections in different control schemes. (b) The normalized cumulative number of infections corresponding to (a). (c-d) The performance of our control scheme under an incomplete network where a fraction of links are randomly removed in the detection process.

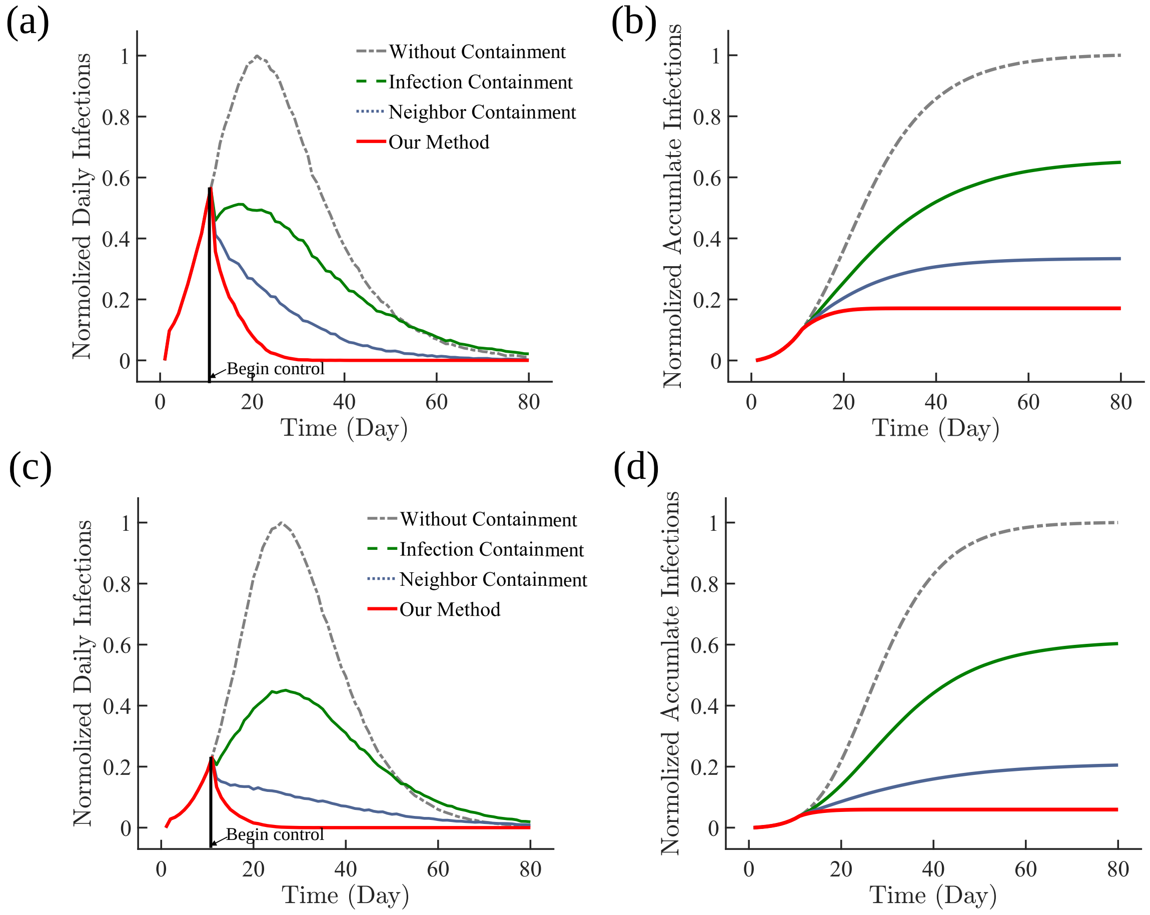

Figure S19: Containment effectiveness of the proposed scheme on simulated COVID-19 spreading with $4\%N$ of individuals in neighbor contain and our method. (a-b) ISA network. (c-d) Email network.

References

[1] 12 more cases discharged, 52 new cases of covid-19 infection confirmed, 2020. https://www.moh.gov.sg/news-highlights/details/12-more-cases-discharged-52-new-cases-of-covid-19-infection-confirmed, Last accessed 4 April 2020.

[2] Links established between church clusters and wuhan travellers, 2020. https://www.moh.gov.sg/news-highlights/details/links-established-between-church-clusters-and-wuhan-travellers, Last accessed 4 April 2020.

[3] R. M. Anderson, B. Anderson, and R. M. May. *Infectious diseases of humans: dynamics and control*. Oxford university press, 1992.

[4] O. Byambasuren, M. Cardona, K. Bell, J. Clark, M.-L. McLaws, and P. Glasziou. Estimating the extent of true asymptomatic covid-19 and its potential for community transmission: systematic review and meta-analysis. *Available at SSRN 3586675*, 2020.

[5] H. C, B. J, and J. T. Covid-19: What proportion are asymptomatic? *Centre for Evidence-Based Medicine*, 2020.

[6] Z. Cao, Q. Zhang, X. Lu, D. Pfeiffer, Z. Jia, H. Song, and D. D. Zeng. Estimating the effective reproduction number of the 2019-ncov in china. *MedRxiv*, 2020.

[7] A. Grover and J. Leskovec. node2vec: Scalable feature learning for networks. In *Proceedings of the 22nd ACM SIGKDD international conference on Knowledge discovery and data mining*, pages 855–864, 2016.

[8] R. Guimera, L. Danon, A. Diaz-Guilera, F. Giralt, and A. Arenas. Self-similar community structure in a network of human interactions. *Physical review E*, 68(6):065103, 2003.

[9] Y. Hu, S. Ji, Y. Jin, L. Feng, H. E. Stanley, and S. Havlin. Local structure can identify and quantify influential global spreaders in large scale social networks. *Proceedings of the National Academy of Sciences*, 115(29):7468–7472, 2018.

[10] N. Imai, I. Dorigatti, A. Cori, C. Donnelly, C. Riley, and N. Ferguson. Report 2: Estimating the potential total number of novel coronavirus cases in wuhan city, china. 22 january 2020-imperial college london. who collaborating centre for infectious disease modelling. mrc centre for global infectious disease analysis, j-idea, imperial college london, uk.

[11] L. Isella, J. Stehlé, A. Barrat, C. Cattuto, J.-F. Pinton, and W. Van den Broeck. What's in a crowd? analysis of face-to-face behavioral networks. *Journal of theoretical biology*, 271(1):166–180, 2011.

[12] T. N. Kipf and M. Welling. Semi-supervised classification with graph convolutional networks. *arXiv preprint arXiv:1609.02907*, 2016.

[13] Q. Li, X. Guan, P. Wu, X. Wang, L. Zhou, Y. Tong, R. Ren, K. S. Leung, E. H. Lau, J. Y. Wong, et al. Early transmission dynamics in wuhan, china, of novel coronavirus–infected pneumonia. *New England Journal of Medicine*, 2020.

[14] T. Liu, J. Hu, M. Kang, L. Lin, H. Zhong, J. Xiao, G. He, T. Song, Q. Huang, Z. Rong, A. Deng, W. Zeng, X. Tan, S. Zeng, Z. Zhu, J. Li, D. Wan, J. Lu, H. Deng, J. He, and W. Ma. Transmission dynamics of 2019 novel coronavirus (2019-ncov). 2020.

[15] M. Majumder and K. D. Mandl. Early transmissibility assessment of a novel coronavirus in wuhan, china. *China (January 23, 2020)*, 2020.

[16] K. Mizumoto, K. Kagaya, A. Zarebski, and G. Chowell. Estimating the asymptomatic proportion of coronavirus disease 2019 (covid-19) cases on board the diamond princess cruise ship, yokohama, japan, 2020. *Eurosurveillance*, 25(10):2000180, 2020.

[17] H. Nishiura, T. Kobayashi, T. Miyama, A. Suzuki, S.-m. Jung, K. Hayashi, R. Kinoshita, Y. Yang, B. Yuan, A. R. Akhmetzhanov, et al. Estimation of the asymptomatic ratio of novel coronavirus infections (covid-19). *International journal of infectious diseases*, 94:154, 2020.

[18] J. M. Read, J. R. Bridgen, D. A. Cummings, A. Ho, and C. P. Jewell. Novel coronavirus 2019-ncov: early estimation of epidemiological parameters and epidemic predictions. *MedRxiv*, 2020.

[19] J. Riou and C. L. Althaus. Pattern of early human-to-human transmission of wuhan 2019 novel coronavirus (2019-ncov), december 2019 to january 2020. *Eurosurveillance*, 25(4):2000058, 2020.

[20] M. Shen, Z. Peng, Y. Xiao, and L. Zhang. Modelling the epidemic trend of the 2019 novel coronavirus outbreak in china. *BioRxiv*, 2020.

[21] B. Tang, X. Wang, Q. Li, N. L. Bragazzi, S. Tang, Y. Xiao, and J. Wu. Estimation of the transmission risk of the 2019-ncov and its implication for public health interventions. *Journal of clinical medicine*, 9(2):462, 2020.

[22] P. Veličković, G. Cucurull, A. Casanova, A. Romero, P. Lio, and Y. Bengio. Graph attention networks. *arXiv preprint arXiv:1710.10903*, 2017.

[23] J. T. Wu, K. Leung, and G. M. Leung. Nowcasting and forecasting the potential domestic and international spread of the 2019-ncov outbreak originating in wuhan, china: a modelling study. *The Lancet*, 395(10225):689–697, 2020.

[24] Y. Yu, Y.-R. Liu, F.-M. Luo, W.-W. Tu, D.-C. Zhan, G. Yu, and Z.-H. Zhou. Covid-19 asymptomatic infection estimation. *medRxiv*, 2020.

[25] S. Zhao, Q. Lin, J. Ran, S. S. Musa, G. Yang, W. Wang, Y. Lou, D. Gao, L. Yang, D. He, et al. Preliminary estimation of the basic reproduction number of novel coronavirus (2019-ncov) in china, from 2019 to 2020: A data-driven analysis in the early phase of the outbreak. *International journal of infectious diseases*, 92:214–217, 2020.

[26] F. Zhou, T. Yu, R. Du, G. Fan, Y. Liu, Z. Liu, J. Xiang, Y. Wang, B. Song, X. Gu, et al. Clinical course and risk factors for mortality of adult inpatients with covid-19 in wuhan, china: a retrospective cohort study. *The lancet*, 2020.
